# Fractionation and characterization of starch granules using field-flow fractionation (FFF) and differential scanning calorimetry (DSC)

**DOI:** 10.1007/s00216-019-01852-9

**Published:** 2019-05-08

**Authors:** Catalina Fuentes, In Kang, Jangjae Lee, Dongsup Song, Malin Sjöö, Jaeyeong Choi, Seungho Lee, Lars Nilsson

**Affiliations:** 1 Food Technology, Engineering and Nutrition, Faculty of Engineering LTH, Lund University, PO Box 124, 221 00 Lund, Sweden; 2 School of Chemistry, Faculty of Pure and Natural Science, Universidad Mayor de San Andres (UMSA), PO Box 330, Cota Cota 27 St., La Paz, 4787 Bolivia; 3 Department of Chemistry, Hannam University, Daejeon, 34054 Republic of Korea

**Keywords:** Starch granule, Gravitational field-flow fractionation (GrFFF), Differential scanning calorimetry (DSC), Split-flow thin cell fractionation (SF)

## Abstract

Starch is one of the main carbohydrates in food; it is formed by two polysaccharides: amylose and amylopectin. The granule size of starch varies with different botanical origins and ranges from less than 1 μm to more than 100 μm. Some physicochemical and functional properties vary with the size of the granule, which makes it of great interest to find an efficient and accurate size-based separation method. In this study, the full-feed depletion mode of split-flow thin cell fractionation (FFD-SF) was employed for a size-based fractionation of two types of starch granules (corn and potato) on a large scale. The fractionation efficiency (*FE*) of fraction-a for corn and potato granules was 98.4 and 99.4%, respectively. The FFD-SF fractions were analyzed using optical microscopy (OM) and gravitational field-flow fractionation (GrFFF). The respective size distribution results were in close agreement for the corn starch fractions, while they were slightly different for the potato starch fractions. The thermal properties of FFD-SF fractions were analyzed, and the results for the potato starch showed that the peak temperature of gelatinization (*T*_p_) slightly decreases as the size of the granules increases. Additionally, the enthalpy of gelatinization (Δ*H*) increases when the granule size increases and shows negative correlation with the gelatinization range (Δ*T*).

**Electronic supplementary material:**

The online version of this article (10.1007/s00216-019-01852-9) contains supplementary material, which is available to authorized users.

## Introduction

Starch is one of the main carbohydrates produced by plants as an energy reserve. It is a mixture of two polysaccharides: amylose and amylopectin, which have different sizes and molar masses. Commonly, the amylose content is 15–35% and the amylopectin 65–85% of the total starch granule. Amylose consists mainly of linear chains of α (1,4)-d-glucose units with some branches in large molecules, while amylopectin has a highly branched structure with α (1–4) linked d-glucose backbones with α (1–6) linked branches [1].

Starch is synthesized in a granular form in special organelles. The biosynthesis of the granule is initiated at the hilum, and the starch granule grows by apposition. The size of the starch granules varies according to their botanical origin, and ranges from less than 1 μm to more than 100 μm in diameter [2, 3]. For example, very small starch granules (i.e., 0.3–2 μm) are present in quinoa, amaranth, and cow cockle; small starch granules (2–10 μm) are found in oats, rice, and buckwheat [2]; medium-size starches (5–30 μm) are found in tapioca, barley, maize, and sorghum [3, 4]; and large starch granules are found in tubers such as potato and canna with sizes of about 100 μm [5]. Furthermore, some types of starches such as potato can have a broad granule size distribution (1 to 110 μm) [6].

It is known that the granule size distribution affects the physicochemical and functional properties of starches [2, 7]. Usually, larger granule fractions show higher amylose contents and lower lipid, protein, and mineral contents and have higher degrees of crystallinity, which might be related to the differences in pasting and thermal properties [7]. Additionally, the peak (*T*_p_) and conclusion (*T*_c_) temperatures of gelatinization increase slightly while the granule size decreases [7–9]. Furthermore, the endothermic enthalpy of gelatinization (∆*H*) decreases when the granule size decreases in the starches [7, 9].

At present, a simple method to separate the starch into different-size fractions is to pass the starch through sieves with different meshes. However, care needs to be taken as the sieving may induce mechanical damage in the starch granules. Another method to fractionate starch is sedimentation [10], but this method is not time efficient for large sample sizes. Therefore, the use of faster and gentler methods for a large-scale starch fractionation would be a good alternative.

Split-flow thin cell fractionation (SF) is an interesting technique that has been shown to be a useful tool for large-scale fractionation of particulate samples with broad size distributions into two subpopulations based on the size or density of the samples [11, 12]. SF has some advantages over other particle fractionation methods, such as static sedimentation and membrane filtration. In SF, samples are not subjected to high mechanical stress during separation. Additionally, SF has a well-constructed theoretical basis, consisting of a “cutoff diameter” that can be easily controlled by adjusting the flow rates [13–15].

There are two operation modes in SF: conventional SF mode and the full-feed depletion SF (FFD-SF) mode. The conventional SF mode has two inlets (for feeding of the sample and the carrier liquid, respectively) and two outlets separated by the flow stream-splitters. In contrast, the FFD-SF mode has only one inlet for sample feeding (inlet-a′) and two outlets (outlets-a, and -b). In addition, an FFD-SF channel could be built in much larger dimensions than a conventional SF channel because it is possible to construct without splitters, allowing substantially higher sample throughput (*TP*) [11, 12].

Another interesting technique is gravitational field-flow fractionation (GrFFF), one of the sub-techniques in the family of field-flow fractionation (FFF) methods, which employs the Earth’s gravity as the external field. GrFFF is relatively simple in principle and easy to operate. GrFFF provides size-based separation of particles with larger ones eluting earlier than smaller ones [16, 17]. GrFFF has been shown to be a useful tool for the separation of various types of micron-sized particles including starch granules, blood cells, yeast, bacteria, and environmental particles [15, 18–26].

In this study, a large-scale FFD-SF was used to separate the corn and potato starch granules into fractions of different sizes. Additionally, GrFFF and optical microscopy (OM) were used to determine the size distributions of the fractions collected from the FFD-SF and differential scanning calorimetry (DSC) was used to evaluate the thermal properties of the FFD-SF fractions of the starch granules.

## Theory

### Full-feed depletion mode of split-flow thin cell fractionation

SF fractionation is based on the size and the density of the samples, which are combined in the definition of cutoff diameter (*d*_c_). In the FFD mode of SF (FFD-SF), *d*_c_ is given by [12, 15]

1 $$ {d}_{\mathrm{c}}=\sqrt{\frac{18\eta }{bLg\Delta  \rho}\left(V\left({a}^{\prime}\right)-V(b)\right)}\kern0.5em $$

where *b* and *L* are the breadth and the length of the FFD-SF channel, respectively; *g* is the value of Earth’s gravity; Δ*ρ* is the difference between the density of the sample and the carrier liquid; *η* is the viscosity of the carrier liquid; and *V*(*a*^′^) and *V*(*b*) are the volumetric flow rates entering inlet-a′ and exiting outlet-b, respectively. In FFD-SF operations, the *d*_c_ and the sample-feeding flow rate *V*(*a*^′^) are chosen first, and then *V*(*b*) is determined using Eq. 1. Once *V*(*b*) is determined, the flow rate exiting outlet-a, *V*(*a*), becomes *V*(*a*^′^) − *V*(*b*). A higher *V*(*a*^′^) allows higher sample throughput (*TP*), which is defined as the mass of the sample that can be processed in a unit time period (g/min or g/h) by [24]

2 $$ TP=V\left({a}^{\prime}\right)\times \mathrm{sample}\ \mathrm{concentration} $$

In this study, fractionation efficiency (*FE*) was defined as the number-percentage of the particles in the FFD-SF fractions having the diameters predicted by theory. *FE* for the FFD-SF fractions-a and -b (*F-a* and *F-b*) were determined by Eqs. 3 and 4, respectively [12].

3 $$ FE\  of\ F-a=\frac{number\ of\le {d}_{\mathrm{c}}\  among\ measured\ particles}{total\ number\ of\ measured\ particles}\times 100\ \left(\%\right) $$

4 $$ FE\  of\ F-b=\frac{number\ of\ge {d}_{\mathrm{c}}\  among\ measured\ particles}{total\ number\ of\ measured\ particles}\times 100\ \left(\%\right) $$

### Gravitational field-flow fractionation

In GrFFF, the retention ratio (*R*) is defined as the ratio of the sample migration velocity to the average velocity of the carrier liquid, and can be measured experimentally by the ratio of the void time (*t*^0^) or the void volume (*V*^0^) to the retention time (*t*_r_) or the retention volume (*V*_r_) as shown in the following equation [16, 27]:

5 $$ R=\frac{V^0}{V_{\mathrm{r}}}=\frac{t^0}{t_{\mathrm{r}}}=3\gamma \frac{d}{w} $$

where *d* is the particle diameter and *w* is the channel thickness. *γ* is the steric correction factor, which can be determined from Eq. 5 by measuring *R* of particles of known diameter. Equation 5 can be rearranged as follows:

6 $$ d=\left(\frac{wt^0}{3\gamma}\right)\left(\frac{1}{t_{\mathrm{r}}}\right)\kern0.5em $$

From Eqs. 5 and 6, *t*_r_ increases as the particle size decreases in GrFFF, providing size-based separation.

Equation 6 allows the determination of *d* from experimental measurements of *t*_r_ if *γ* is known. Since *γ* varies with experimental conditions, a calibration curve is usually used for the determination of *d* from measurements of *t*_r_. Rearranging Eq. 6, we obtain the following expression:

7 $$ \log\ {t}_{\mathrm{r}}=-{S}_d\log d+\log A $$

where *S*_*d*_ is the size-based selectivity and *A* is the extrapolated *t*_r_ of unit *d* of the particles [28].

In general, plots of log*t*_r_ vs. log*d* are used as a calibration plot for size analysis by GrFFF and, in turn, for conversion of GrFFF fractograms to mass-based size distributions. Additionally, GrFFF-UV fractograms can be converted to number-based size distributions by the following equation [29, 30]:

8 $$ number\  PSD=\left(\frac{UV\  response}{particle\ volume}\right)/\left(\sum \frac{UV\  response}{particle\ volume}\right) $$

## Materials and methods

### Materials

Potato starch (PS, 03967) and corn starch (PS, S4126) having granular sizes ranging 2–100 μm and 2–30 μm determined by OM, respectively, were purchased from Sigma-Aldrich (St. Louis, MO, USA). The densities of the corn and potato starch granules were determined to be 1.34 and 1.22 g/mL, respectively. The determination of density was performed from the experimental results of FFD-SD using Eq. 1. The size of fraction-a was determined by OM, then the highest size observed in this fraction was used as *d*_c_ and the density was re-calculated. The carrier liquids used for FFD-SF and GrFFF were deionized water from a Milli-Q system (Millipore Co. Ltd., Billerica, USA; resistance = 18.2 MΩ/cm), either without added chemicals for FFD-SF or with addition of 0.1% (*w*/*v*) FL-70 (Fisher Chemical, NJ, USA) and 0.02% (*w*/*v*) NaN_3_ (Sigma-Aldrich, St. Louis, USA) for GrFFF.

Polyurethane (PU) latex bead (DMX-400CY, Soken, Japan) was used to test the FFF-SF system before applying it to starch samples. Polystyrene (PS) latex beads (Duke Scientific, *ρ* = 1.05 g/mL, Palo Alto, CA, USA) with nominal diameters of 6, 8, 12, 20, and 50 μm were used to create the calibration curve (log*t*_r_vs. log *d*) for size determination of the starch granules by GrFFF. The calibration curve had good linearity with *R*^2^ = 0.998 (see Electronic Supplementary Material (ESM) Fig. S1). Although the analyzed starch granules are not spherical, the calibration curve is obtained from analysis of spherical PS particles. In a previous study, it has been shown that the calibration remains valid up to an aspect ratio of 12 [31]. Hence, as the analyzed starch granules in the current study have a maximum aspect ratio of 3, the calibration procedure is considered valid.

### Methods

#### Sample preparation

Each starch sample was mixed with pure Milli-Q water at a concentration of 3.0 g/L, and stirred for at least 2 h until it was well dispersed at room temperature (i.e., about 25 °C). For each starch sample, 10 L of dispersion was prepared in total.

Potato starch has a wide granular size distribution ranging from 2 to about 100 μm, and some of the larger granules sediment quickly on the bottom of the FFD-SF channel. Potato starch granules larger than 50 μm were removed using static sedimentation before FFD-SF separation. The sedimentation time was determined using Eq. 9

9 $$ \varDelta t=\frac{18\eta \varDelta x}{\varDelta \rho {d}_c^2g} $$

where *η* is the dynamic viscosity of the carrier liquid in which the sample is dispersed; ∆*x* is the difference between the initial and final distance where the sedimentation takes place, i.e., the sedimentation height; Δ*ρ* is the difference between the density of the sample and the carrier liquid; *d*_c_ is the cutoff diameter (the size limit between fractions); and *g* is the gravitational acceleration (9.8 m/s^2^). After the defined sedimentation time, the suspension was collected. Milli-Q water was added to the remaining sediment and dispersed again. This sedimentation process was repeated five times, and the sediment potato starch granules (> 50 μm) were dried at room temperature until the weight was constant. The dried sediment was weighed to determine the final concentration of the potato starch granule dispersion (granules < 50 μm). The final concentration of the potato starch dispersion was 2.76 g/L.

#### Large-scale FFD-SF and GrFFF

**Fig. 1:**
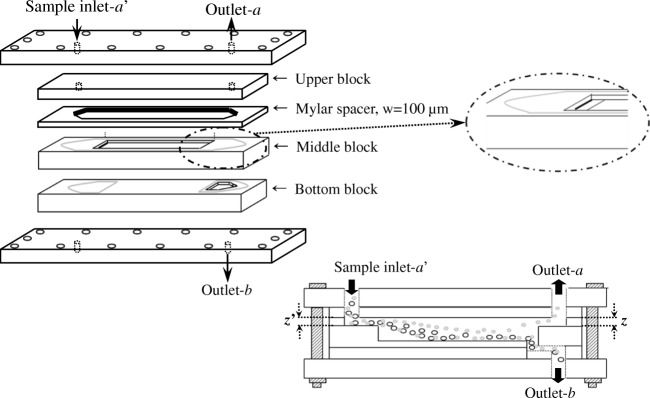
Channel assemblies of the large-scale FFD-SF used in this study [12]

A large-scale FFD-SF channel was assembled without splitters for this study, as shown in Fig. 1. The channel, from the top to the bottom, consisted of an upper block, a Mylar spacer, a middle block, and a bottom block. As shown, the total channel length was 30 cm with a width of 6 cm, and thickness of 1700 μm including 100 μm of the Mylar spacer [11, 12]. A peristaltic pump (LEPP 150 L, Labscitech, Corona, CA, USA) was used to feed the sample through inlet-a′, and the two outlet flow rates (*V*(*a*) and *V*(*b*)) were adjusted by connecting tubing of various diameters and lengths to the outlets.

The FFD-SF fraction collected from outlet-b (fraction-b) was fed again through inlet-a′ to improve the *FE* of fraction-b. This process was repeated three times for each sample. After FFD-SF fractionation, GrFFF and OM were used to determine the size distribution for the analysis of each collected fraction. For the DSC measurement, each FFD-SF fraction was dried at room temperature until the weight was constant before the measurements.

The GrFFF channel was cut in a 200-μm-thick Mylar spacer, which was placed between two glass plates and clamped between two acryl blocks. The breadth and length of the GrFFF channel were 2 and 50 cm, respectively. The carrier liquid was pumped by an HPLC pump (SP930D, Young-Lin, Seoul, Korea) at a flow rate of 1.0 mL/min. The elution of the sample was monitored by a UV detector (M720, Young-Lin, Seoul, Korea), set at a wavelength of 254 nm. The samples were injected directly into the channel using a 100-μL syringe (Hamilton Co., Reno, NV, USA) through a rubber septum at a flow rate of 0.2 mL/min. After the injection, the sample was allowed to settle across the channel thickness by stopping the channel flow for relaxation of the sample. The sample was eluted on resumption of the flow. All GrFFF analyses were performed in triplicate to ensure reproducibility.

#### Optical microscopy

The OM (Olympus BX51TF, Shinjuku Monolith, Japan) was used to measure the number-based size distributions of the starch granules. For each sample, about 500 starch granules were observed and measured using the Image Inside™ software (Focus, Daejeon, Korea). For OM analysis of the starch granules of irregular shapes, the longest dimensions of the granules were taken as diameters.

#### Differential scanning calorimetry

The gelatinization properties were determined by differential scanning calorimetry (DSC) using differential scanning calorimeter 6200 (Seiko Instruments, Shizuoka, Japan) over the temperature range 10 to 150 °C with a scanning rate of 10 °C/min. The samples were prepared in the same way as described elsewhere in excess water (1:3 *w*/*v*) [32]. Thermo-gravimetric curves were recorded on the EXSTAR 6000 Thermal Analysis System equipped with the Muse Standard Analysis Software, version 6.4 (SII Nano Technology Inc., Chiba, Japan). The onset (*T*_o_), peak (*T*_p_), and conclusion (*T*_c_) temperatures and enthalpy of gelatinization on starch dry weight basis (Δ*H*) were determined. The measurements were performed in triplicate.

## Results and discussion

### Size fractionation by a large-scale FFD-SF

The large-scale FFD-SF channel was assembled as described above. The starch granule samples were continuously fed through inlet-a′ while stirring, in order to prevent the starch granules from settling. Both the corn and the potato starch were separated into two fractions (fraction-a and fraction-b, respectively). There was an additional fraction of the potato starch that was obtained from pre-fractionation by sedimentation (fraction-c). The cutoff diameters *d*_c_ set for FFD-SF separation of the corn and the potato starch granules were 15 and 30 μm, respectively, and the outlet flow rates *V*(*a*) and *V*(*b*) were 45 and 55 mL/min for the corn starch and 120 and 180 mL/min for the potato starch, respectively (Eq. 1).

**Fig. 2:**
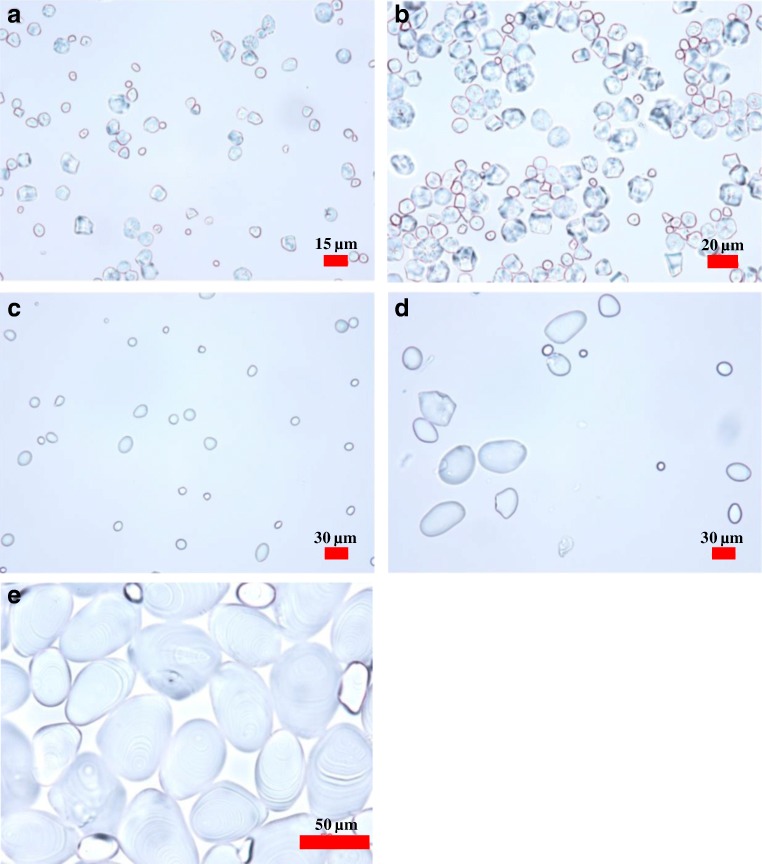
Optical microscopy (OM) images of corn and potato starch granule fractions. Corn starch granules, **a** fraction-a and **b** fraction-b from FFD-SF; potato starch granules, **c** fraction-a and **d** fraction-b from FFD-SF, and **e** fraction-c from static sedimentation

**Fig. 3:**
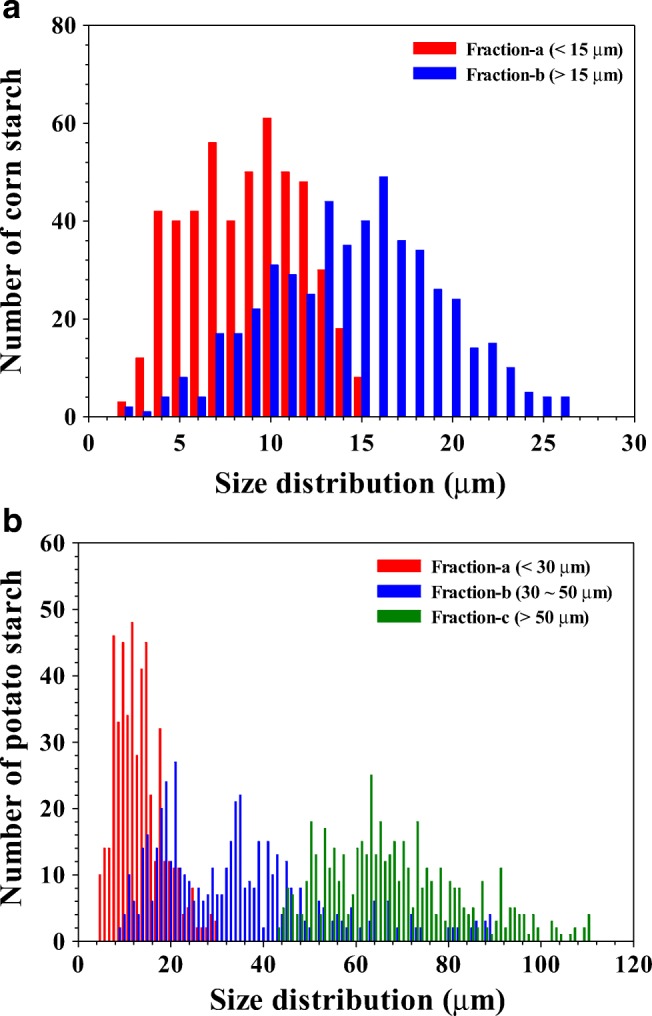
Number-based size distribution of **a** corn starch granules and **b** potato starch granules obtained by optical microscopy (OM)

Figure 2 shows the OM images of the corn (a and b) and potato (c, d, and e) starch granule fractions. The number-based size distributions of the fractions shown in Fig. 2 are depicted in the chart in Fig. 3. The shapes of the corn starch granules were polygonal with slightly dented faces, while the potato starch granules had oval or ellipsoid shapes which become more remarkable as the size increases as shown in Fig. 2.

The *FE*s calculated by Eq. 3 for fraction-a of the corn and potato starch granules were 98.4 and 99.4%, respectively, which indicates that FFD-SF separations of starch granules smaller than the *d*_c_ value (15 and 30 μm) were excellently achieved. The *FE*s calculated by Eq. 4 for fraction-b of the corn and potato starch granules were only 52.2 and 45.4%, respectively. Thus, the *FE* values for the fraction-b’s were significantly lower than those for the fraction-a’s. This was somewhat expected by the absence of inlet-b′ in the FFD mode [11, 12, 33, 34].

In order to increase *FE* in fraction-b, the collected fraction-b was fed again through inlet-a′, yielding “fraction-ba” and “fraction-bb.” This was repeated three times in total, yielding “fraction-bba,” “fraction-bbb,” “fraction-bbba,” and “fraction-bbbb.” At the end, fractions-a, -ba, -bba, and -bbba were mixed together to make “total fraction-a.” Table 1 shows the summary of *FE* and the average size (*d*_avg_) measured for each fraction. The number-based size distributions obtained by OM for all FFD-SF fractions of the corn and potato starch granules are shown in the ESM (Figs. S2 and S3, respectively).

**Table 1:** Fraction efficiency (*FE*) and average size (*d*_avg_) of FFD-SF fractions of corn and potato starch granules

Type of starch	Corn (*TP* = 18 g/h, *d*_avg_ = 13.4 μm)		Potato (*TP* = 49.7 g/h, *d*_avg_ = 37.7 μm)	
	*FE* (%)	*d*_avg_ (μm)	*FE* (%)	*d*_avg_ (μm)
Fractiona	98.4	8.6	99.4	13.5
Fraction-ba	97.0	8.9	98.8	13.6
Fraction-bba	94.8	9.3	97.6	16.8
Fraction-bbba	95.8	9.6	98.2	17.5
Total fraction-a	96.5	9.1	98.5	15.3
Fraction-b	52.2	14.6	45.4	33.9
Fraction-bb	63.6	15.8	54.6	36.8
Fraction-bbb	84.2	17.2	71.0	42.7
Final fraction-bbbb	92.6	17.8	84.0	49.0

Table 1 shows that the *FE* of fractions-b increased gradually as the FFD-SF separation was repeated, and reached 92.6% and 84.0% for corn and potato starch, respectively, after repeating three times. The *d*_avg_ of fractions-b also increased gradually as the FFD-SF separation was repeated, probably due to an increase in the *FE*. For fractions-a, no particular trend was observed, and the final *FE*s measured for “total fraction-a” were over 95% for both starch samples, suggesting that the FFD-SF separations were successfully performed according to the *d*_c_ set for each sample.

Figure 3 shows that fraction-b of potato starch contained granules larger than 50 μm, and fraction-c contained granules smaller than 50 μm. This indicates that no complete separation by the static sedimentation to remove granules larger than 50 μm was achieved. Nevertheless, fraction-c still showed a good *FE* value: 92.2%. This incomplete separation of fraction-c did not significantly affect the FFD-SF separation of potato starch for granules smaller than 50 μm since the final *FE* of fraction-bbbb was higher than 80%. Furthermore, no starch granules > 35 μm were observed in fractions-a. Detailed results on the size distributions and *FE*s obtained by OM for all FFD-SF fractions are shown in the ESM (Fig. S3).

### Size characterization by GrFFF

**Fig. 4:**
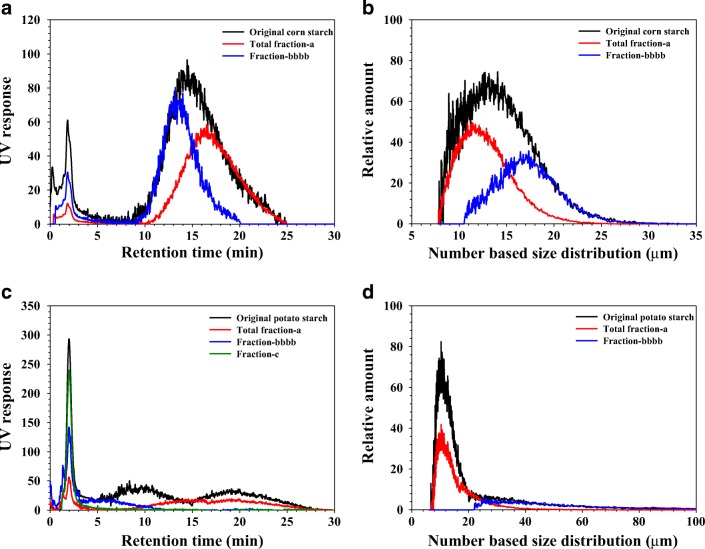
GrFFF fractograms (**a**) and number-based size distributions (**b**) of the original (unfractionated sample) and total fraction-a and fraction-bbbb of corn starch. GrFFF fractograms (**c**) and number-based size distributions (**d**) of the original (unfractionated sample) and total fraction-a, fraction-bbbb, and fraction-c of potato starch

Total fraction-a, fraction-bbbb, and for potato starch fraction-c were further analyzed by GrFFF. Figure 4 shows the GrFFF fractograms and number-based size distributions of the original (whole sample, i.e., unfractionated sample) and the fractions of corn and potato starch samples.

In the case of corn starch (Fig. 4a, b), the size ranges and the average size (*d*_avg_) of the original, total fraction-a, and fraction-bbbb were determined by Eq. 8 to be 7 to 30 (12.9) μm, 7 to 25 (11.9) μm, and 11 to 30 (16.2) μm, respectively. The *d*_avg_ values from OM and GrFFF are in reasonable agreement (see Table 2).

**Table 2:** Average size (*d*_avg_) from OM and GrFFF and thermal properties measured at a starch-to-water ratio of 1:3 (*w*/*v*) for starch fractions

Sample		*d*_avg_ from OM (μm)	*d*_avg_ from GrFFF (μm)	*T*_o_ (°C)	*T*_p_ (°C)	*T*_c_ (°C)	*T*_c_–*T*_o_ (°C)	Δ*H* (mJ/mg) based on dry matter
Corn starch	Original	13.4	12.9	64.1 ± 0.3	70.0 ± 0.1	74.6 ± 0.1	10.5 ± 0.2	9.2 ± 0.2
	Total fraction-a	9.1	11.9	64.4 ± 0.3	70.0 ± 0.1	74.6 ± 0.3	10.5 ± 0.2	9.1 ± 0.2
	Fraction-bbbb	17.8	16.2	64.6 ± 0.2	70.1 ± 0.1	74.3 ± 0.3	9.7 ± 0.4	8.9 ± 0.2
Potato starch	Original	37.7	Not measured	60.7 ± 0.2^a^	65.1 ± 0.2^a^	70.2 ± 0.6^a^	9.6 ± 0.5^a^	14.9 ± 0.7^a^
	Total fractiona	15.3	12.3	61.5 ± 0.6^b^	66.7 ± 0.2^b^	72.5 ± 0.2^b^	10.9 ± 0.7^b^	13.0 ± 0.4^b^
	Fraction-bbbb	49.0	39.8	61.9 ± 0.2^b^	65.9 ± 0.1^c^	70.8 ± 0.4^a^	8.9 ± 0.4^a^	16.8 ± 0.2^c^
	Fractionc	68.1	Not measured	61.6 ± 0.1^b^	65.6 ± 0.1^d^	70.4 ± 0.1^a^	8.8 ± 0.3^a^	17.1 ± 0.2^c^

Mean values ± standard deviation (*n* = 3). Values with the same letter within the same column are not significantly different. Significance was determined using Tukey’s test *α* = 0.05 and the following critical mean differences: *T*_o_ = 0.8 °C, *T*_p_ = 0.4 °C, *T*_c_ = 1.0 °C, *T*_c_–*T*_o_ = 1.3 °C, and ∆*H* = 1.1 mJ/mg

*T*_*o*_ onset temperature, *T*_*p*_ peak temperature, *T*_*c*_ conclusion temperature, *T*_*c*_*–T*_*o*_ gelatinization range, *ΔH* enthalpy of gelatinization

It seems, in the case of potato starch, that some of the granules in the original (whole sample) and fraction-c are so large that they are eluted without retention with the void peak (see Fig. 4c), for which the size distributions were not measured. For total fraction-a and fraction-bbbb, the size ranges and average sizes (*d*_avg_) were determined to be 7 to 40 (12.3) μm and 23 to 100 (39.8) μm, respectively (Fig. 4d).

For fraction-bbbb of the potato starch, *d*_avg_ determined from GrFFF is about 10 μm smaller than that from OM. This may be because, for OM analysis of a starch granule of irregular shape, the longest dimension of the granules was taken as the diameter. In GrFFF, as shown by Eq. 6, the size of a particle is determined from its retention time (or the migration velocity of the particle in the GrFFF channel), which depends on the average distance of the particle from the bottom of the channel [17, 27, 35], not on the longest dimension of the particle. In the ESM (Fig. S4), the number-based size distributions obtained from OM and GrFFF for fraction-a and fraction-bbbb of the corn and potato starch are shown.

### Thermal properties of starch granules: effect of size

The thermal properties of the original and total fraction-a, fraction-bbbb, and fraction-c of corn and potato starch, respectively, are shown in Table 2. For corn starch, no significant differences were observed between the original sample and the total fraction-a and fraction-bbbb in the measured thermal properties.

To determine if original potato starch and its fractions were different from a statistical point of view, Tukey’s test based on triplicate analysis was applied, which showed *T*_o_ of the original sample (i.e., whole potato starch sample) (60.7 °C) was significantly different from those of the fractions, and there were no significant differences among the fractions. Tukey’s test also showed that the *T*_p_ values of the original potato starch and its fractions were significantly different from each other. In addition, *T*_p_ decreased slightly (from 66.7 to 65.6 °C) with increasing granule size (from < 30 to > 50 μm), which is in agreement with two previous studies [9, 10].

Singh et al. fractionated potato starch granules from four different cultivars. The starch suspension in pure water was passed sequentially through 200-mesh and 500-mesh sieves (i.e., 75 μm and 30 μm), obtaining three fractions. The first fraction with granule size < 30 μm had *T*_p_ = 62.2–64.4 °C. The second fraction with granule size 74–30 μm had *T*_p_ = 61.8–63.7 °C, and the third fraction with granule size ≥ 75 μm had *T*_p_ = 60.3–62.1 °C, showing a slight decrease in *T*_p_ with increasing granule size [9].

Dhital et al. fractionated potato starch granules by sedimentation, and the fractions were obtained by decanting the suspension that remained after a specific time. The method is described in detail elsewhere [10]. Five fractions: very small (VS), small (S), medium (M), large (L), and very large (VL) were obtained with surface-weighted mean diameters of 15.9, 28.1, 40.2, 50.5, and 67.5 μm determined by light diffraction, and *T*_p_ values of 63.5, 63.0, 62.9, 62.6, and 62.2 °C, respectively, were found. [7]. The results showed a slight decrease in *T*_p_ with increasing granule size. Additionally, fractions S and M were not significantly different from each other and similarly for L and VL. VS was significantly different from the rest of the fractions, determined by analysis of variance (*p* ≤ 0.05).

In the current study, the *T*_c_ of total fraction-a (72.5 °C) was significantly different from those of the other fractions. *T*_c_ decreased as the size of the granule increased (70.4 °C for fraction-bbbb), and was similar for granules larger than 30 μm (70.8 °C for fraction-c). As there was no significant difference in *T*_o_, fraction-bbbb and fraction-c had a similar gelatinization temperature range (Δ*T* = *T*_c_–*T*_o_). Δ*H* increased from 13.0 to 17.1 mJ/mg when the granule size increased, which means that there was a negative correlation between gelatinization temperature ranges and Δ*H*. These results are in agreement with the results reported by Dhital et al. [7], as described above, where Δ*H* is in the range of 14.9 to 16.7 mJ/mg and increases when the granule size increases and Δ*H* has negative correlation with Δ*T* that is in the range of 14.0 to 10.4 °C.

It has been reported that thermal properties of starch vary with the granule size which in turn could be influenced by several factors such as botanical source [2]; mineral, lipid, protein, and amylose content; and the amount of double- and single-helical structures [36]. In literature, it has been reported that amylose content could have a strong impact. One reason is the direct relation between amylose content and thermal properties and their relation with granule size [7, 37, 38]. It has been shown that higher amylose content is present in larger starch granules [9, 38–40]. In addition, high-amylose starches have somewhat higher gelatinization temperatures [41, 42], which suggests that such granules could be more resistant to thermal gelatinization and swelling.

## Conclusion

In this study, a large-scale FFD-SF was employed for size-based fractionation of the corn and potato starch granules. Prior to FFD-SF, granules larger than 50 μm were removed from the potato starch sample using static sedimentation. The results show that a large-scale FFD-SF is useful for separation of starch granules with *d*_c_ lower than about 50 μm. The *FE*s of fraction-a were excellent (higher than about 98%) for both samples. In order to improve the *FE* of fraction-b, the fraction collected from outlet-a was re-fed through inlet-a′ three times. In addition, OM and GrFFF are shown to be suitable methods for the determination of the size distribution of the granules. The results from OM and GrFFF for the corn starch granules were in close agreement, while those for the potato starch granules were slightly different due to the non-spherical (ellipsoidal) shapes of the granules. It was also shown that the peak temperature of gelatinization, *T*_p_, decreases slightly as the size of the starch granules increases. Additionally, the enthalpy of gelatinization (Δ*H*) increases when the granule size increases and Δ*H* has a negative correlation with gelatinization range Δ*T* = *T*_c_–*T*_o_, showing that the thermal properties vary with the granule size, which could be related to the amylose content.

## Electronic supplementary material

ESM 1 (PDF 1074 kb)
