# Supplementary material for: Fractionation and characterization of starch granules using field-flow fractionation (FFF) and differential scanning calorimetry (DSC)
Source: Anal Bioanal Chem. 2019 May 8;411(16):3665–74. doi: 10.1007/s00216-019-01852-9 (PMC6571092; doi:10.1007/s00216-019-01852-9)
Supplement: Supplementary file 1 — (PDF 1074 kb) [file 216_2019_1852_MOESM1_ESM.pdf]

Analytical and Bioanalytical Chemistry

Electronic Supplementary Material

Fractionation and characterization of starch granules using field-flow fractionation (FFF) and differential scanning calorimetry (DSC)

Catalina Fuentes, In Kang, Jangjae Lee, Dongsup Song, Malin Sjöö, Jaeyeong Choi, Seungho Lee, Lars Nilsson

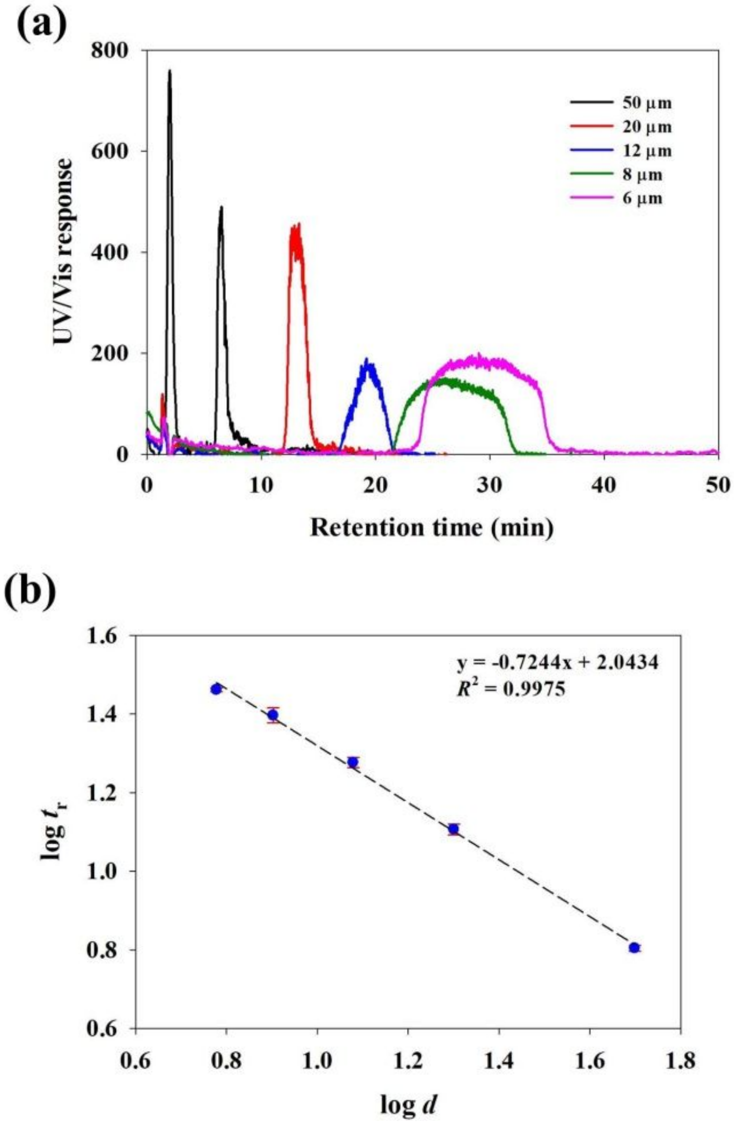

Fig. S1 GrFFF fractograms of polystyrene latex beads having nominal diameters of 6, 8, 12, 20 and 50 µm, respectively (a) and a calibration curve (b)

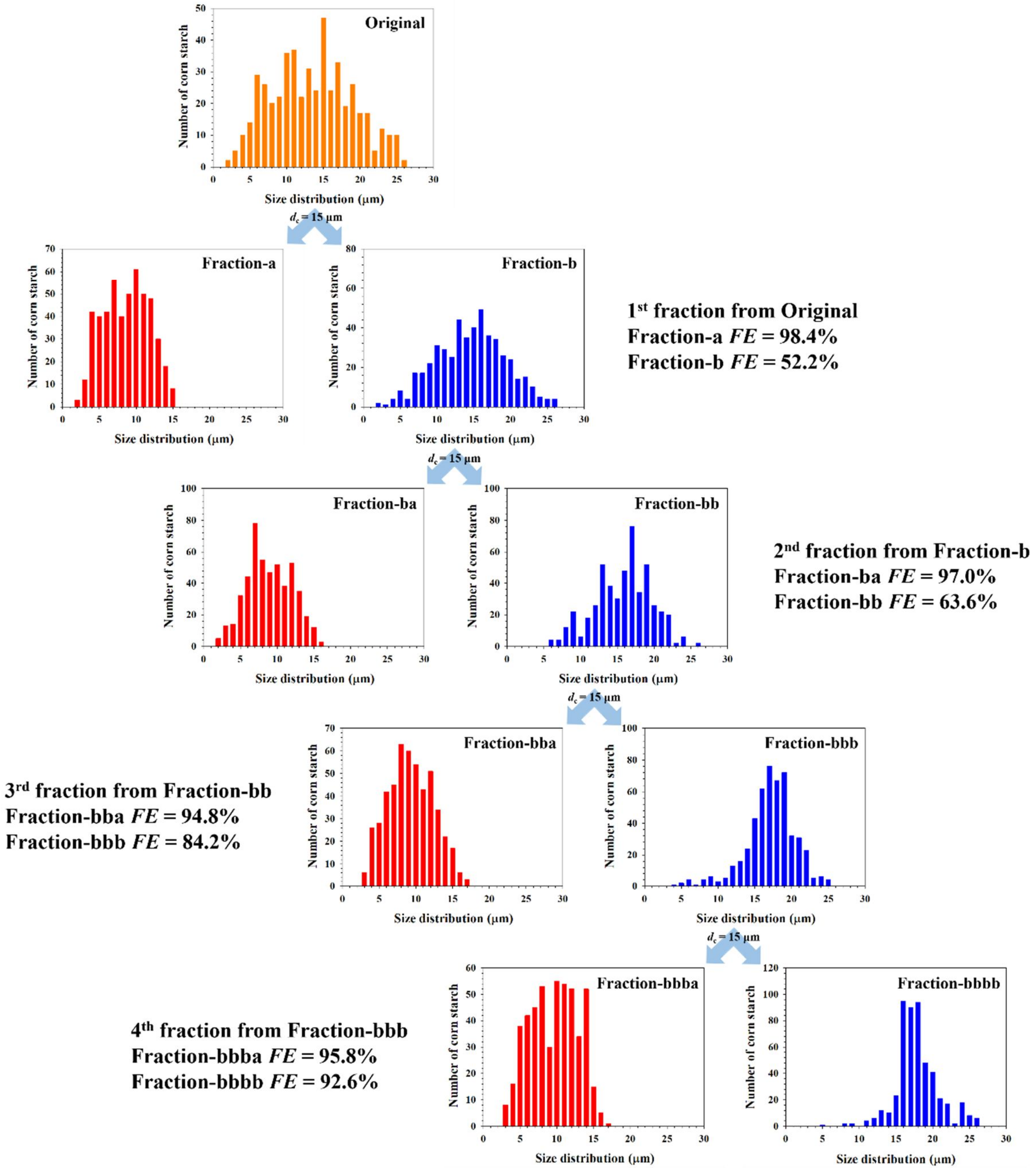

Fig. S2 Number based size distributions and *FE*’s from OM obtained for FFD-SF fractions of corn starch, n=500

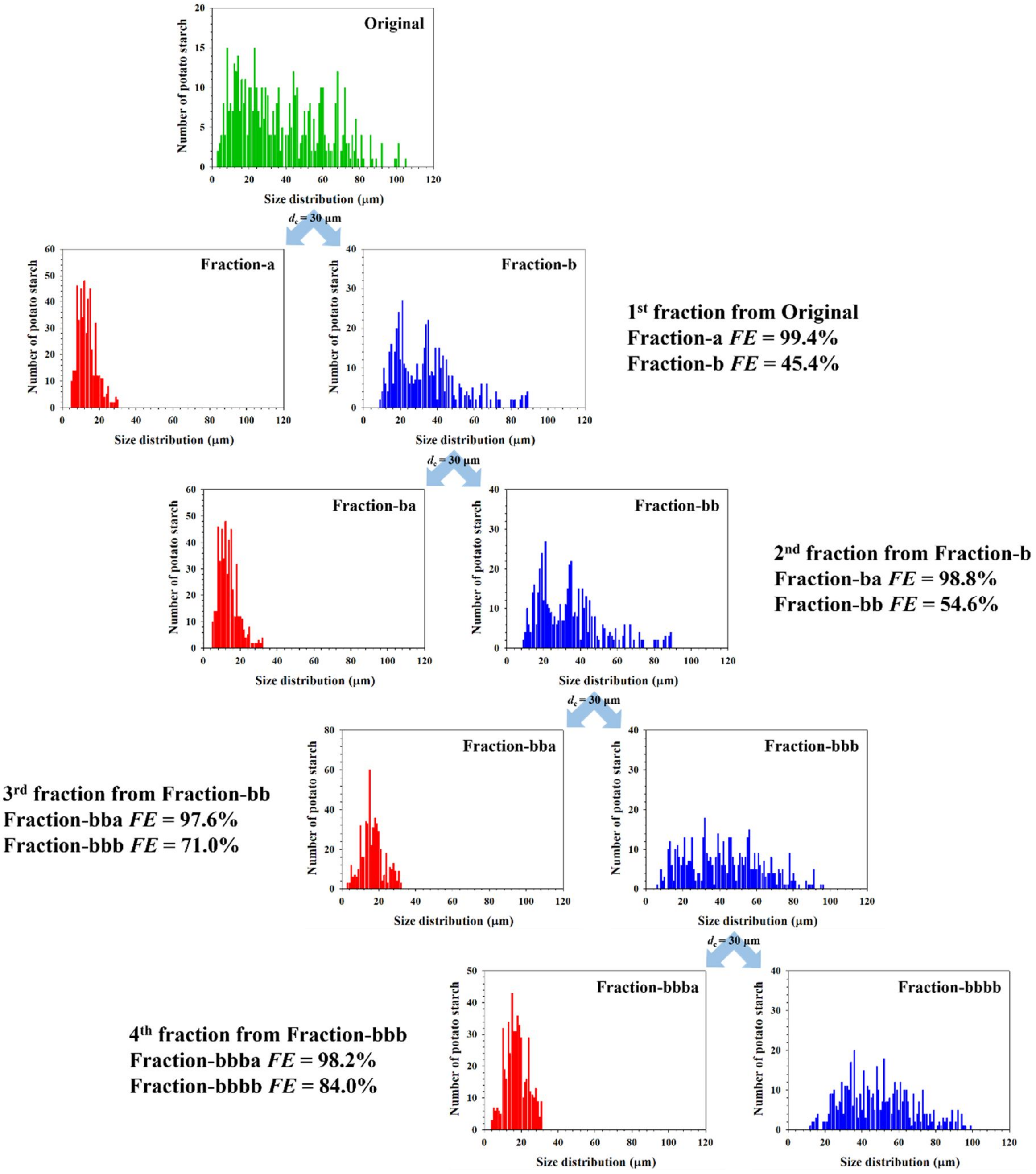

Fig. S3 Number based size distributions and *FE*'s from OM obtained for FFD-SF fractions of potato starch, n=500

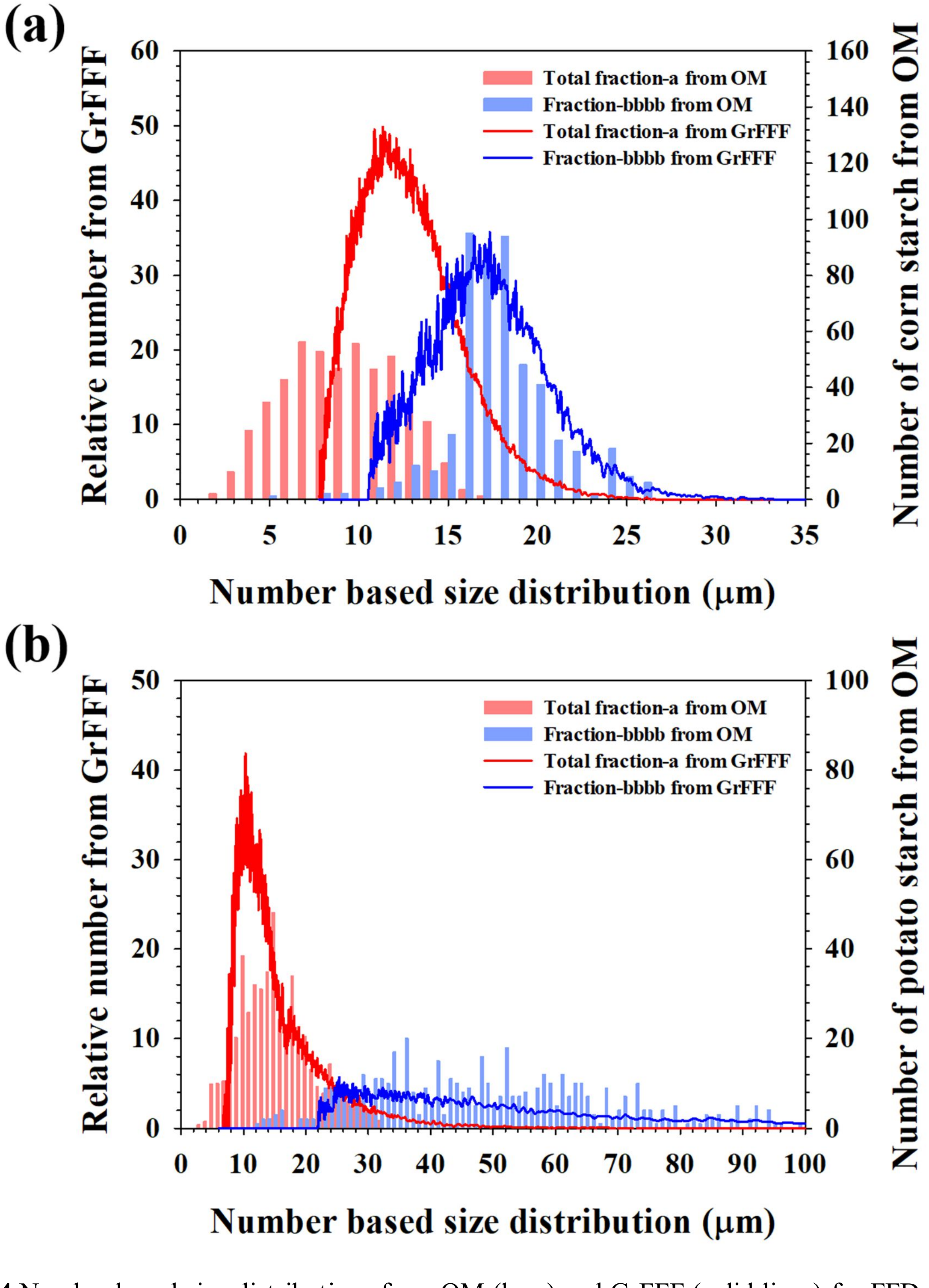

Fig. S4 Number based size distributions from OM (bars) and GrFFF (solid lines) for FFD-SF fractions of corn (a) and potato starch (b)
